# Prenatal Exposure to Diethylstilbestrol and Multigenerational Psychiatric Disorders: An Informative Family

**DOI:** 10.3390/ijerph18199965

**Published:** 2021-09-22

**Authors:** Marie-Odile Soyer-Gobillard, Laura Gaspari, Françoise Paris, Nicolas Kalfa, Samir Hamamah, Philippe Courtet, Charles Sultan

**Affiliations:** 1 Arago Laboratory, CNRS, University Sorbonne, 75016 Paris, France; elido66@orange.fr; 2 Association HHORAGES-France, 95270 Asnières-sur-Oise, France; 3 Service de Pédiatrie, Unité d’Endocrinologie-Gynécologie Pédiatrique, CHU Montpellier, University Montpellier, 34090 Montpellier, France; laura.gaspari@inserm.fr (L.G.); f-paris@chu-montpellier.fr (F.P.); 4 Constitutif Sud, Centre de Référence Maladies Rares du Développement Génital, Hôpital Lapeyronie, CHU Montpellier, University Montpellier, 34090 Montpellier, France; nicolaskalfa@gmail.com; 5 INSERM 1203, Développement Embryonnaire Fertilité Environnement, University Montpellier, 34295 Montpellier, France; s-hamamah@chu-montpellier.fr; 6 Département de Chirurgie Viscérale et Urologique Pédiatrique, Hôpital Lapeyronie, CHU Montpellier, University Montpellier, 34090 Montpellier, France; 7 Institut Debrest de Santé Publique IDESP, UMR INSERM, University Montpellier, 34090 Montpellier, France; 8 Biologie de la Reproduction/DPI et CECOS, Département de Biologie de la Reproduction, CHU Montpellier, University Montpellier, 34090 Montpellier, France; 9 Département d’Urgence et Post Urgence Psychiatrique, CHU Montpellier, University Montpellier, 34090 Montpellier, France; p-courtet@chu-montpellier.fr

**Keywords:** psychiatric disorders, diethylstilbestrol (DES), multigenerational transmission, prenatal exposure, epigenetic

## Abstract

Background: Psychiatric disorders in children exposed in utero to diethylstilbestrol (DES) are still debated. We report here the impact of DES prescribed to suppress lactation on the children born after such treatment and their progeny, focusing particularly on psychiatric disorders. Case presentation: We report here an informative family in which one or more psychiatric problems (e.g., bipolarity, suicide attempts and suicide, eating disorders) were detected in all children of second-generation (DES-exposed children; *n* = 9), but for II-2 who died at the age of 26 years due to rupture of a congenital brain aneurysm, and were associated with non-psychiatric disorders (particularly, endometriosis and hypospadias). In the third generation, 10 out of 19 DES-exposed grandchildren had psychiatric disorders (autism spectrum disorder, bipolar disorder, dyspraxia and learning disabilities, mood and behavioral disorders, and eating disorders), often associated with comorbidities. In the fourth generation (7 DES-exposed great-grandchildren, aged between 0 and 18 years), one child had dyspraxia and autism spectrum disorder. The first daughter of the second generation (not exposed to DES) and her children and grandchildren did not have any psychiatric symptoms or comorbidities. Conclusions: To our knowledge, the high prevalence of psychiatric disorders of various severities in two, and likely three generations, including DES-free pregnancies and DES-exposed pregnancies from the same family, has never been reported. This work strengthens the hypothesis that in utero exposure to DES contributes to the pathogenesis of psychiatric disorders. It also highlights a multigenerational, and possibly transgenerational, effect of DES in neurodevelopment and psychiatric disorders.

## 1. Introduction

Diethylstilbestrol (DES) was synthetized by Dodds in 1938, and has been widely used as therapeutic estrogen to prevent miscarriages, inhibit lactation, and to treat many hormonal disorders [1]. The urogenital effects (e.g., female genital abnormalities, vaginal cancer, and male urogenital disorders) of DES exposure during fetal life have been well documented for several decades. Conversely, the occurrence of psychiatric disorders following in utero exposure is still debated. The review by Kebir and Krebs included only three large epidemiological studies that reported the occurrence of psychiatric disorders in DES children and seven studies on a small number of exposed patients [2]. Specifically, the American Nurses’ Health Study I on two generations (treated pregnant mothers and exposed daughters) found an increased risk of depression during adulthood in the 1612 DES daughters [3]. Psychiatric disorders were also detected in a cohort of 1002 DES children from a French national patient association (HHORAGES-France) and in a national French survey on DES daughters [4,5,6].

Here, we report the presence of psychiatric disorders and comorbidities in an informative family where the mother (first generation) took DES to inhibit lactation after each pregnancy. These findings suggest that prenatal exposure to DES might affect neurodevelopment, with a possible multigenerational (and likely transgenerational) effect on psychiatric disorder occurrence.

## 2. Informative Family

**Figure 1:**
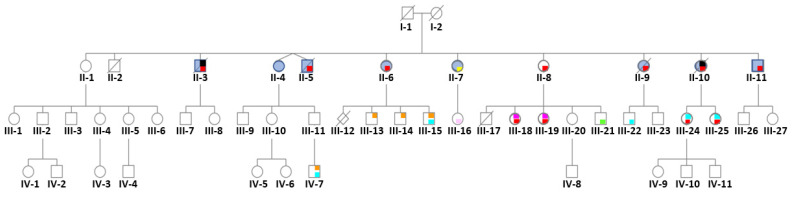
Pedigree of the informative family whose mother (I-2) was treated with DES (30 mg/day) for 3 months after each delivery to inhibit lactation. Only the first child (II-1) was not exposed to DES in utero. Daughter II-1 and her descendants do not have any psychiatric disorders. No history of psychiatric disorders was reported for the maternal and paternal sides. Legend: red = bipolar disorder, yellow = psychosis borderline, blue = attempted suicide(s), black = suicide, orange = autism spectrum disorder, light blue = dyspraxia and learning disabilities, light pink = mood and sleep disorders, green = behavioral disorders, fuchsia = eating disorders.

We report on a single family of Caucasian origin with two (and likely three) generations affected by psychiatric disorders (see pedigree, Figure 1). The index case (II-7) joined the French association of DES-treated women (HHORAGES Association), reporting psychiatric disorders and laparoscopically confirmed endometriosis. The family history revealed that other family members had psychiatric disorders often associated with other diseases, such as endometriosis. The diagnoses of psychiatric disorders were performed following the Diagnostic and Statistical Manual of Mental Disorders criteria after hospitalization of the concerned family members in a neuropsychiatric ward, after consultation with skilled clinicians, and/or with use of standardized and validated diagnostic instruments, and then verified in all family members by the medical record or a physician’s note given by the witness (II-7). Endometriosis was diagnosed as previously described [7].

The mother (I-2) from Canada did not have any psychiatric symptoms. She worked as waitress and then as nurse until retirement. The father (I-1) worked as miller and then primary school teacher until retirement. They had 11 full-term children (one twin pregnancy). The mother (I-2) was thus gravida/para/abortion (GPA) G10 P11 A0. The mother (I-2) underwent surgery (three times) for recurrent benign breast tumors, had severe Graves-Basedow disease, and died at the age of 80 years due to vascular dementia complications. She had two half-sisters, and the father (I-1) had three brothers and three sisters. None of them displayed symptoms of psychiatric disorders according to the witness.

DES (30 mg/day per os) was systematically prescribed, by the same gynecologist, to the mother for voluntary suppression of puerperal lactation for aesthetic reasons for 3 months after each delivery, starting from the first one. Medication adherence was complete. Therefore, the first child (II-1), a daughter without any history of psychiatric problems, was not prenatally exposed to DES because treatment was started after her birth. All other children of the second generation (except II-2, who died at the age of 26 years due to rupture of a congenital brain aneurism) developed psychiatric disorders (Figure 1, Table 1). Specifically, II-3 and II-10 had bipolar disorder, alcohol dependence, a history of suicide attempts, and died due to suicide at the age of 42 (II-3) and 50 (II-10). II-5, II-7, II-8, II-9 and II-11 had bipolar disorder, alcohol dependence, and a history of suicide attempts. II-4 and II-7 had eating disorders, and II-6 bipolar disorder and a history of suicide attempts.

Nine of them also had other health disorders, such as uvula bifida (II-2), deafness of the left ear (II-3), endometriosis (II-4, II-6, II-7, II-8, II-9, II-10), and multiple cancers (II-7).

In the third generation (Figure 1 and Table 1), psychiatric disorders were diagnosed in 10 of the 19 surviving children (III-6: anencephalic fetus and III-17: very premature child who died at day 3 post-partum) of DES daughters: Autism Spectrum Disorder (ASD) with Asperger syndrome (III-13 and III-14), and ASD with dyspraxia (III-15) (three sons of daughter II-6); mood and sleep disorders (III-16, daughter of II.7); bipolar and eating disorders (III-18 and III-19) and behavioral disorders (III-21) (daughters and son, respectively, of II.8); dyspraxia (III-22 son of II.9); and bipolar disorder and learning disabilities (III-24 and III-25; daughters of II.10). Moreover, nine of them also had endometriosis (*n* = 7) or hypospadias (*n* = 2).

In the fourth generation (age ranging from 0 to 18 years), ASD and dyspraxia were diagnosed in boy IV-7 (12 years of age).

None of the children and grandchildren of the only unexposed daughter (II-1) had psychiatric symptoms. Moreover, no occupational or environmental exposure to endocrine disrupting chemicals was recorded, except for III-25 (esthetician).

## 3. Discussion

In this informative family, several prenatally DES-exposed children and their descendants had psychiatric illnesses, except the only unexposed first daughter and her progeny. The heritability estimates for most psychiatric disorders have been found to be high, between 0.4 and 0.8, demonstrating the role of genetic factors in the etiology of mental illness [8]. We were unfortunately unable to collect blood DNA from this family to investigate single nucleotide gene polymorphisms. Nevertheless, the absence of any psychiatric disorders, either personally or in their extended family, in the parents as well as in the first unexposed daughter and her progeny indirectly suggests the key role of environmental influences. To our knowledge, this high prevalence of psychiatric disorders of various severities in two, and likely three generations, including DES-free pregnancies and DES-exposed pregnancies from the same family, has never been reported.

The mother (I-2) was systematically prescribed DES (30 mg/day per os for 3 months) to inhibit lactation after each delivery. The frequency of DES prescription to stop lactation after a delivery has not been estimated yet, and to our knowledge, no study has reported its impact on children born after such treatment. Earlier studies on DES pharmacodynamics in mammals found that DES concentration could be quantified in the liver at day 120 after oral intake of ^14^C-DES [9]. As DES disposition in humans is similar to what is observed in animal models, in the case of I-2, the subsequent pregnancies might have begun when DES was still present in the body, because the interval between gestations was between 3 and 8 months. Whether each fetus might have been exposed to the residual DES constitutes a potential limitation. Moreover, DES is mainly metabolized into its catechol and then quinone that reacts with DNA to form adducts stored in the adipose tissue [10]. Quinones can have deleterious neurodevelopmental effects by inducing severe modifications of single- and double-stranded DNA during their metabolization, and also transplacental genotoxic effects [11,12]. Moreover, they can induce genotoxic DNA adducts, possibly in several generations, thus reinforcing the hypothesis of a multigenerational (and likely transgenerational) transmission of DES-linked psychiatric disorders in this informative family [13,14].

In a large-scale study (Nurses’ Health Study I), O’Reilly et al. demonstrated a marked increase of depressive disorders in DES children exposed in utero [3]. Psychiatric disorders (particularly bipolarity, schizophrenia) have been documented in children exposed in utero to DES or ethinylestradiol in the HHORAGES-France patient association [2,4]. Similarly, a case-control epidemiological study in a large Chinese population found that 11% of the 235 identified cases of ASD occurred in children conceived while taking oral contraceptives that were continued during early pregnancy [15]. Baron-Cohen et al. demonstrated in a Danish cohort a clear link between higher concentration of estrogen and synthetic progestins in the amniotic fluid and development of ASD-type disorders in the children later in life [16]. These results support the hypothesis by Strifert on the link between oral contraceptives and ASD prevalence in children [17]. Accordingly, in animal studies, Zou et al. demonstrated that prenatal exposure to the estrogen–progestogen combination or to progestins induces autism-like behavior in young rats [18].

In the present work, comorbidities were mainly detected in the reproductive organs (endometriosis and hypospadias), as previously described [7,19]. This is in agreement with the molecular genomic and epigenomic study by Rivollier et al., which demonstrated that psychiatric disorders in DES children are correlated with specific methylome modifications in two genes: *ZFP57*, implicated in neurodevelopment, and *ADAM-TS*, involved in uterine and reproductive organ development, and probably playing a critical role in the control of central nervous system development with effects on neurodevelopment and neuroplasticity [20,21,22].

Regarding the multigenerational (and likely transgenerational) DES effect in human diseases, few studies focused on the association between DES exposure and the occurrence of male genital malformations and cervical clear cell carcinoma in DES grandchildren [19,23]. The only report on DES-linked psychiatric effects in DES grandchildren recently published by Kioumourtzoglou et al. included 47,540 participants spanning three generations, and concluded that DES exposure is associated with multigenerational neurodevelopmental disorders. Our report is in agreement with this work [24].

Regarding ASDs more specifically, it has been hypothesized that they originate in utero due to molecular perturbations of the developing brain. For instance, Ladd-Acosta et al. in a pilot post-mortem study found that *ZFP57* is more methylated in cerebellar tissue samples of female patients with autism than unrelated controls [25]. Moreover, in a post-mortem study, Corley et al. showed significant DNA methylation defects in the brain, supporting the hypothesis of an early developmental origin of ASD [26]. In Sprague–Dawley rats, Zou et al. detected DNA hyper-methylation on the promoter of the gene encoding the estrogen receptor β in the amygdala of the offspring in utero exposed to estro-progestogens or to progestogens [18]. Interestingly, this effect was less important in female than male rats, as observed also in humans, in agreement with our observations.

In this family, a direct causal link between the mother’s treatment with DES and the development of psychiatric disorders in all children of the first generation prenatally exposed to DES and most of their subsequent descendants cannot be demonstrated, and thus remains speculative. Whether there would be enough DES remaining in the body to affect these pregnancies cannot be determined, which is a limitation. However, the analysis of this informative family suggests that prenatal exposure to DES promotes the development of psychiatric disorders and reinforces the suspicion of a multigenerational (and likely transgenerational) effect of DES in human disease. The presence of comorbidities, mainly endometriosis and hypospadias, associated with psychiatric disorders is a signature of the already demonstrated effects of DES exposure.

## Figures and Tables

**Table 1:** Psychiatric disorders (columns 2–3) and comorbidities (column 4) diagnosed in the members of the informative family (column 1) whose mother took DES for 3 months after each delivery (therefore, II-1 was not exposed): 11 children (second generation: II-1 to II-11), born from 1956 to 1971, 27 grandchildren (third generation: III-1 to III-27) born from 1979 to 2004, and 11 great-grandchildren (fourth generation: IV-1 to IV-11) born from 2002 to 2020. The diagnosed psychiatric disorders are listed in column 3. In the second generation, eight children had bipolar disorders and a history of suicide attempts and/or completed suicide (columns 5 and 6), and two had eating disorders. Nine children also had comorbidities. In the third generation, three boys had ASD, and four girls had bipolar disorder. Among the great-grandchildren, one boy (born in 2008) has ASD and dyspraxia without any comorbidities. ♂: male; ♀: female;/: too young to be diagnosed.

Patient	Psychiatric Disorder	Diagnosis	Associated Non-Psychiatric Disorders	Suicide Attempt(s)	Death by Suicide
II-1 ♀	no	no	no	no	no
II-2 ♂	no	no	Uvula bifida, death due to ruptured congenital brain aneurysm at 26 years of age	no	no
II-3 ♂	yes	Bipolar disorder, chronic alcoholism	Deafness of left ear	yes	yes (at 42 years of age)
II-4 (♀ twin)	yes	Eating disorder	Endometriosis	no	no
II-5 (♂ twin)	yes	Bipolar disorder, chronic alcoholism	Chronic cirrhosis death at 51 years of age	yes	no
II-6 ♀	yes	Bipolar disorder	Endometriosis, inverted kidney/bladder	yes	no
II-7 ♀	yes	Bipolar disorder, chronic alcoholism, eating disorder	Endometriosis, sacrococcygeal teratoma, rectal adenocarcinoma, breast cancer	yes	no
II-8 ♀	yes	Bipolar disorder, chronic alcoholism	Endometriosis, fibromyalgia, obesity	yes	no
II-9 ♀	yes	Bipolar disorder, chronic alcoholism	Endometriosis	yes	no
II-10 ♀	yes	Bipolar disorder, chronic alcoholism	Endometriosis	yes	yes (at 50 years of age)
II-I1 ♂	yes	Bipolar disorder, chronic alcoholism	No	yes	no
III-1 ♀	no	no	no	no	no
III-2 ♂	no	no	no	no	no
III-3 ♂	no	no	no	no	no
III-4 ♀	no	no	no	no	no
III-5 ♀	no	no	no	no	no
III-6 ♀	no	no	no	no	no
III-7 ♂	no	no	no	no	no
III-8 ♀	no	no	no	no	no
III-9 ♂	no	no	no	no	no
III-10 ♀	no	no	Endometriosis	no	no
III-11 ♂	no	no	Hypospadias	no	no
III-12 ♂	/	Very premature baby, anencephaly, deceased	/	/	/
III-13 ♂	yes	ASD, Asperger syndrome	Hypospadias	no	no
III-14 ♂	yes	ASD, Asperger syndrome	no	no	no
III-15 ♂	yes	ASD, learning disorder (dyspraxia)	no	no	no
III-16 ♀	yes	Mood and sleep disorders	Endometriosis	no	no
III-17 ♂	/	Very premature baby, (deceased at day 3 post-partum)	/	/	*/*
III-18 ♀	yes	Bipolar disorder, eating disorders	Endometriosis	no	no
III-19 ♀	yes	Bipolar disorder, eating disorders	Endometriosis	no	no
III-20 ♀	no	no	Endometriosis	no	no
III-21 ♂	yes	Behavioral disorders	no	no	no
III-22 ♂	yes	Learning disorder (dyspraxia)	no	no	no
III-23 ♂	no	no	no	no	no
III-24 ♀	yes	Bipolar disorder, learning disorder	Endometriosis	no	no
III-25 ♀	yes	Bipolar disorder, learning disorder	Endometriosis	no	no
III-26 ♂	no	no	No	no	no
III-27♀	no	no	no	no	no
IV-1 ♀	no	no	no	no	no
IV-2 ♂	/	/	/	/	/
IV-3 ♀	no	no	no	no	no
IV-4 ♂	/	no	no	no	no
IV-5 ♀	no	no	no	no	no
IV-6 ♀	no	no	no	no	no
IV-7 ♂	yes	ASD, learning disorder (dyspraxia)	no	no	no
IV-8 ♂	/	/	/	/	*/*
IV-9 ♀	/	/	/	/	*/*
IV-10 ♂	/	/	/	/	*/*
IV-11 ♂	/	/	/	/	*/*

## Data Availability

No new data were generated or analyzed in support of this research.

## Note

The association HHORAGES-France, a patient family association, is registered at the Epidemiological Portal of French Health Databases INSERM (French National Institute for Medical Research) and AVIESAN (National Alliance for Life Sciences and Health) (epidemiologiefrance.aviesan.fr accessed on 22 July 2021).
